# Towards 4D Printing of Very Soft Heterogeneous Magnetoactive Layers for Morphing Surface Applications via Liquid Additive Manufacturing

**DOI:** 10.3390/polym14091684

**Published:** 2022-04-21

**Authors:** Lucas Brusa da Costa Linn, Kostas Danas, Laurence Bodelot

**Affiliations:** Solid Mechanics Laboratory (LMS), CNRS, École Polytechnique, Institut Polytechnique de Paris, 91128 Palaiseau, France; lucas.linn02@gmail.com (L.B.d.C.L.); konstantinos.danas@polytechnique.edu (K.D.)

**Keywords:** 4D printing, liquid additive manufacturing, magnetorheological elastomers, magnetoactive layers, morphing surface, silicone composite, very soft materials

## Abstract

This work explores the use of liquid additive manufacturing (LAM) to print heterogeneous magnetoactive layers. A general method is proposed where, by studying the printing of pure silicone lines, the successful printing of closed shapes, open shapes, and a combination thereof, can be achieved while accounting for the continuous deposition that is specific to LAM. The results of this characterization are subsequently exploited for the printing of a heterogeneous layer composed of four magnetoactive discs embedded in a pure silicone square. Such a layer, when affixed to a softer silicone substrate, yields a system that produces truly three-dimensional surface patterns upon application of a magnetic field. Hence, this work demonstrates that LAM is a promising approach for the rapid 4D printing of morphing surfaces exhibiting 3D surface patterns that can be actuated remotely and reversibly via a magnetic field. Such heterogenous layers have a wide range of applications, ranging from haptics to camouflage to differential cell growth.

## 1. Introduction

Magnetoactive polymers are made of magnetizable particles dispersed in a polymeric matrix. The mechanical properties of the polymer matrix can range from very soft (in the case of gels and elastomers), to soft (in the case of acrylate polymers), to hard (in the case of thermosets). Once the magnetic field is removed, the employed particles can also be classified as soft-magnetic particles if they exhibit low remanence or hard-magnetic particles if they exhibit significant remanence [1,2,3]. According to the selected combination of matrix and particles, the obtained magnetoactive composites exhibit different behaviors. In particular, very soft matrices with soft-magnetic particles—also known as magnetorheological elastomers (MREs)—undergo magnetostriction (magneto-induced deformation) as the particles seek to align along the lines of the applied magnetic field [4,5,6,7,8]. Note that this behavior is accompanied with a concomitant change in stiffness, which has been largely studied for tunable damping applications [9,10]. All matrices with soft magnetic particles, when shaped as elongated bodies, are subject to the compass effect and exhibit large deflections under an applied magnetic field [10,11,12]. Finally, soft and hard matrix layers containing hard magnetic particles whose magnetization axis has been carefully oriented in space throughout the layer (or which contain soft particles that were aligned in chains along the magnetic field during curing) can exhibit complex motions such as waving, crawling, jumping or rolling when torques are generated for orienting the magnetization axes of the particles (or the axis of the particle chains) with the applied magnetic field [13,14,15,16,17]. Such behaviors, especially because they are triggered in an untethered fashion via magnetic fields, naturally lend themselves to applications in remote actuation, soft robotics and medical devices [2,18,19,20].

As with most polymeric materials, magnetoactive polymers were initially fabricated by molding, which exhibits significant drawbacks: each desired object requires the manufacturing of a specific mold, and some shapes cannot be attained due either to limitations in the achievable mold geometry or to limitations in the demolding process. Hence, with the development of 3D printing [21,22], there has been an increasing amount of work dedicated to the 3D printing of magnetoactive polymers, which by essence falls under the scope of 4D printing [23]. Hard matrices are exclusively printed by fused filament fabrication (FFF) and can be embedded either with hard magnetic particles [24] for permanent magnet applications or with soft magnetic particles [25,26] that are usually used as a heat source when submitted to a high-frequency magnetic field in order to trigger a shape recovery in the printed shape-memory polymers. With the recent broadening of materials available for FFF, Cao et al. [27] printed bio-inspired soft actuators with a thermoplastic rubber filament loaded with carbonyl iron particles (CIPs). Soft matrices are usually printed using photolithography techniques such as digital light processing (DLP), [14,16,28,29,30] or by direct ink writing (DIW) [31,32]. In the case of DIW, even if a very soft silicone is used as the initial pure matrix, the addition of magnetic particles in addition to the rheology-modifying fillers (added to obtain an ink that exhibits the DIW-required shear-thinning properties) yields a final composite that is much harder that the host matrix. In most studies, very soft materials were not necessary since the focus was in controlling the magnetic axis orientation in the composite to leverage displacements induced by the compass effect. Additionally, the printed objects were composed of a single magnetoactive blend [33,34,35,36,37] and, so far, only Bastola et al. [38,39] have proposed multimaterial 3D printing in the context of MREs by embedding high-viscosity magnetorheological fluid patterns in a silicone matrix via DIW. The printing of very soft polymers that do not exhibit any shear-thinning properties (which thereby falls out of the scope of DIW) is known as liquid additive manufacturing (LAM). It remains a rather marginal topic and has been only addressed so far in the case of non-functional materials (i.e., pure silicone) [40,41,42,43] with applications in biomedicine or pneumatic-based soft robotics. More recently, German RepRap (now innovatiQ) [44] has introduced a commercial printer for the 3D printing of liquid silicone rubber, though the proposed compatible material has a rather high viscosity (~150,000 cps) and lies in the “harder” range for very soft silicones (50 Shore A).

**Figure 1:**
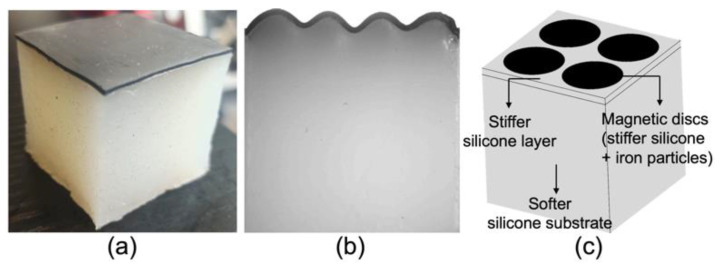
(**a**) System made of a stiffer MRE layer (Ecoflex 00-50 and 20 vol% CIP, thickness 0.8 mm) bonded to a softer pure silicone substrate. The whole system measures 40 mm × 40 mm × 40 mm and corresponds to the one used in [48]. (**b**) A 2.5D wrinkling pattern via collaborative instabilities, obtained when this system is submitted to a lateral compression of 20% and a 0.4 T magnetic field perpendicular to the layer. (**c**) Schematic of the heterogeneous magnetoactive layer targeted in this study, comprising of a set of four magnetoactive discs (17 mm in diameter) embedded in a non-magnetic square (40 mm × 40 mm in size) and fused to a soft non-magnetic substrate.

To the best of our knowledge, LAM has never been employed for printing functional silicone blends, nor for combining silicones with different properties. In this work, the objective is to exploit LAM to print a very soft heterogenous magnetoactive layer without the additional rheology-modifying (but non-functional) fillers that would be required by DIW and that would ultimately harden the resulting blend. The considered layer comprises a set of four magnetoactive discs (17 mm in diameter) embedded in a non-magnetic square (40 mm × 40 mm in size). This heterogeneous layer is subsequently fused onto an even softer non-magnetic cubic silicone substrate (see Figure 1c), and the obtained system is expected (and demonstrated here) to exhibit three-dimensional reversible patterns at its surface under remote magnetic actuation. Hence the broader goal of this work is to address the generation of truly three-dimensional patterns in magneto-activated morphing surfaces for potential applications in haptics or differentiated cell growth. As a matter of fact, morphing surfaces based on magneto-activated layers have been so far limited to rather simple shapes. In particular, most examples consist of circular layers clamped at their edge [45,46,47] that produce a simple concave or convex shape under magnetic actuation. More complex surface patterns in the form of periodic wrinkles (Figure 1b) have been obtained by combining mechanical and magnetic instabilities in a system made of a thin and rather stiff MRE uniform layer bonded to a thick and soft non-magnetic substrate [48,49] (see Figure 1a). Nevertheless, such patterns can only be considered as 2.5D patterns since they correspond to a profile extruded in one direction. Thus, reaching controllable targeted (truly) 3D patterns in the context of magnetic field actuation has remained elusive.

In this context, a promising avenue is to leverage LAM to print layers exhibiting spatially heterogeneous magnetic particle contents. To achieve this, it is first necessary to study the printing properties of silicone in order to determine what influence the printing speed, the extrusion speed, the bed temperature, and the elapsed printing time (due to the temporal change of viscosity) have on the printed silicone. In parallel, it is also necessary to study the influence of the syringe needle diameter and initial silicone viscosity on the final dimensions of the printing. Once the silicone printing behavior is understood (Section 3.1), the printing of geometric shapes can be addressed (Section 3.2). Since the targeted MRE surface shown in Figure 1c is a silicon square with embedded magnetic discs, it is necessary to determine which shape printing parameters, such as infill pattern and infill density, allow these geometric shapes to be obtained without over- or under-extrusion. Finally, the printing of the heterogeneous magnetoactive layer and its bonding to a soft, non-magnetic substrate are discussed in Section 3.3, and the behavior of the obtained system under a magnetic field is assessed both experimentally and numerically in Section 3.4.

## 2. Materials and Methods

### 2.1. D Printer

**Figure 2:**
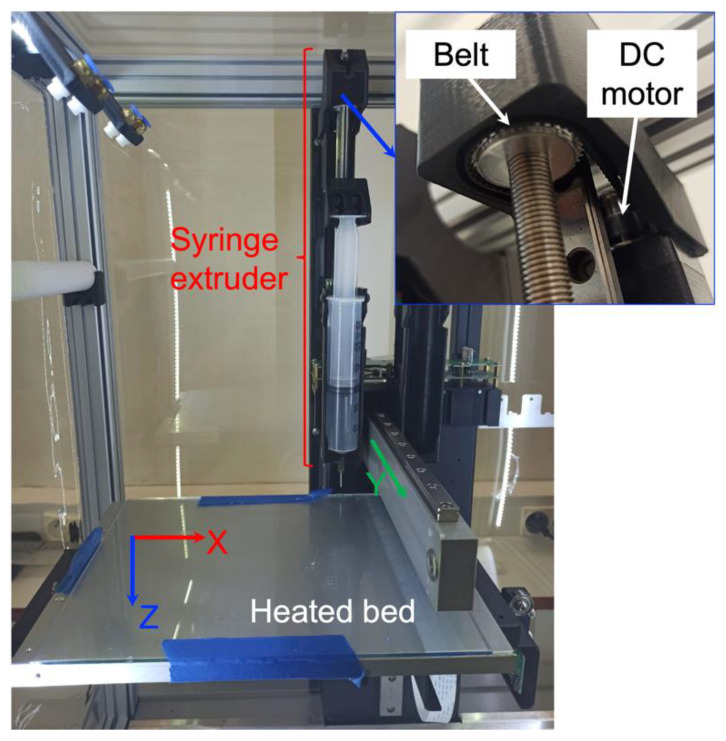
The Hyrel System 30 M 3D printer used in this work.

In this study, a Hyrel System 30 M printer (Hyrel 3D, Norcross, GA, USA) with SDS 60 syringe extruders was used (Figure 2). The printer possesses three degrees of freedom, since the tray (or bed) can move horizontally in the X direction and in the vertical Z direction, while the syringe holder can move horizontally in the Y direction. The building tray can also be heated up to 90 °C. The vertical movement of the syringe piston is controlled by a DC motor so that the rotation of the motor is transferred to the syringe piston holder via a belt (see inset in Figure 2). The rotation of the motor, and thereby the extrusion speed, can be controlled via G-code commands (see Section 2.4).

### 2.2. Syringes

BD (Franklin Lakes, NJ, USA) Plastipak^TM^ 50–60 mL syringes were used with the SDS 60 extruders. Flat-end needles were mounted at the tip of the syringes to perform the printings. In order to assess the influence of the diameter of the needle, two diameters were used: 1.6 mm and 0.84 mm (14 gauge and 18 gauge, respectively).

### 2.3. Silicones, Particles, and Sample Preparation

Four different silicone blends from Smooth-On (Macungie PA, USA, often employed in soft robotics applications) have been used to determine the influence of viscosity on the printing. All silicones were room-temperature vulcanization (RTV) silicones and were composed of two components, A and B, that needed to be mixed, where A is the silicone and B is the platinum-based catalyst causing the silicone to cure. Note that the curing process can be accelerated by increasing the temperature. The properties of the employed silicones are listed in Table 1.

In the case of the magnetoactive blend, carbonyl iron powder SM from BASF (Ludwigshafen, Germany) was employed. It was composed of spherical particles of 3.5 μm median diameter that are magnetically soft, since they do not retain magnetization once the magnetic field is turned off.

Before printing, the preparation of the pure silicone samples consisted of 5 main steps: (1) pouring Part B into Part A, (2) manual mixing for 30 s, (3) degassing for 4 min in a vacuum chamber, (4) feeding the syringe with the silicone blend, and (5) loading the syringe into the printer.

The preparation of the magnetoactive silicone samples included two additional preliminary steps before adding Part B: (0a) pouring Part A on top of the particles, and (0b) manual mixing for 30 s.

Concerning the fourth step of the sample preparation process, feeding the syringe with the silicone blend could be carried out in two ways: by pouring or by suction. These two forms were tested, and it was observed that feeding the syringe by suction led to a large number of bubbles in the printed body (see Figure S1) as air was mixed again with the degassed silicone during the suction. On the contrary, feeding by pouring did not result in bubbles and was thus employed throughout this study.

However, with feeding by pouring, when the plunger of the syringe was placed, the silicone started flowing spontaneously. Hence, there was a deposition of material in unwanted places due to the subsequent continuous flow of silicone by gravity. In the case of feeding by suction, there was no such spontaneous flow. Nevertheless, with this form of feeding, it was still very difficult to precisely control the deposition of material, since both the triggering of flow by the lowering of the syringe piston and the interruption of flow by the retraction of the piston did not occur instantly (resulting in unwanted material deposition as well). Note that the rheology of the silicone can be modified to prevent spontaneous extrusion by gravity and to better control the flow of material out of the needle by the piston, although this is the objective of DIW [31]. The goal of this work is to assess the behavior of both pure and magnetoactive silicones during printing as a function of their intrinsic parameters without the addition of other fillers (which is the essence of LAM).

### 2.4. Software

The 3D printer is controlled by the Repetrel firmware (4.1 version, Hyrel 3D, Norcross, GA, USA). To carry out the prints, it was necessary to directly input in Repetrel the G-code that commands the printer in terms of tool path and printing parameters. Thus, the part to be printed was first modeled using a computer-aided design (CAD) software (here, SolidWorks 2019, Dassault Systèmes, Vélizy-Villacoublay, France), and converted into an STL file. The latter was then imported into a slicer software (here, Slic3r, 1.3 version, open-source software) in order to generate the tool path based on user-defined printing parameters for the infill pattern and the infill density. Other printing parameters such as printing speed, extrusion speed, and bed temperature were also embedded in the G-code file generated by the slicer software.

## 3. Results and Discussion

### 3.1. Printing Properties of Silicone Lines

#### 3.1.1. Influence of Printing Speed, Extrusion Speed, and Bed Temperature on the Final Line Width

To determine the influence of a parameter on the silicone printing, the tests were carried out while keeping all the other parameters constant and varying only the parameter being analyzed. Thus, in all tests studying the influence of the printing parameters, the silicone used was fixed as Ecoflex 00-20 and the syringe needle diameter was fixed as 1.6 mm.

**Figure 3:**
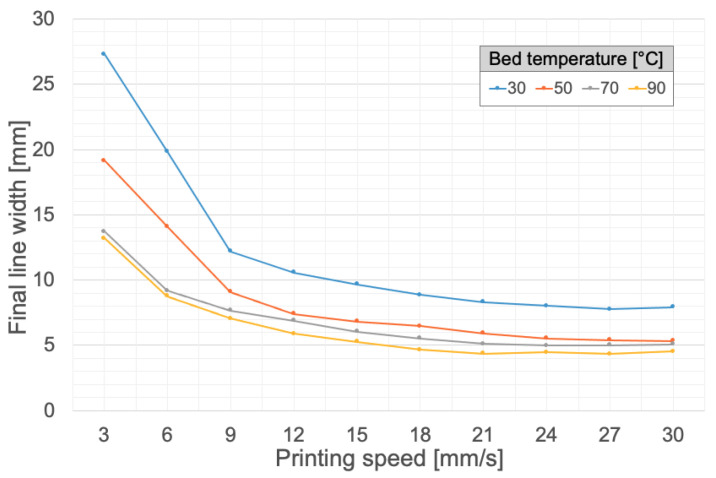
Influence of bed temperature and printing speed on the final line width. Silicone: Ecoflex 00-20. Needle diameter: 1.6 mm. Extrusion speed: 0.05 mm/s.

In particular, for determining the influence of printing speed and bed temperature on the final width of the lines, a series of lines was printed at fixed extrusion speed and fixed temperature, where the printing speed was reduced at each line from 30 mm/s to 3 mm/s by 3 mm/s increments (as illustrated in Figure S2). The widths of the printed lines were measured using the software ImageJ (open-source software, after determining the conversion ratio between pixels and millimeters). The test shown in Figure S2 was conducted for a fixed extrusion speed of 0.05 mm/s and for temperatures of 30 °C, 50 °C, 70 °C, and 90 °C, thus yielding the results reported in Figure 3.

It can be seen in Figure 3 that, the higher the temperature, the smaller the final width of the printed line. As a matter of fact, a higher temperature means a faster cure, which prevents the silicone from spreading further on the printing bed surface. In addition, a greater printing speed leads to a smaller printed line width since it translates into a shorter silicone deposition time, thereby resulting in a smaller final width. For a printing speed of 30 mm/s, the line width reaches a minimum regardless of the printing temperature. At such a speed, the difference in width between 50 °C, 70 °C and 90 °C becomes minimal.

**Figure 4:**
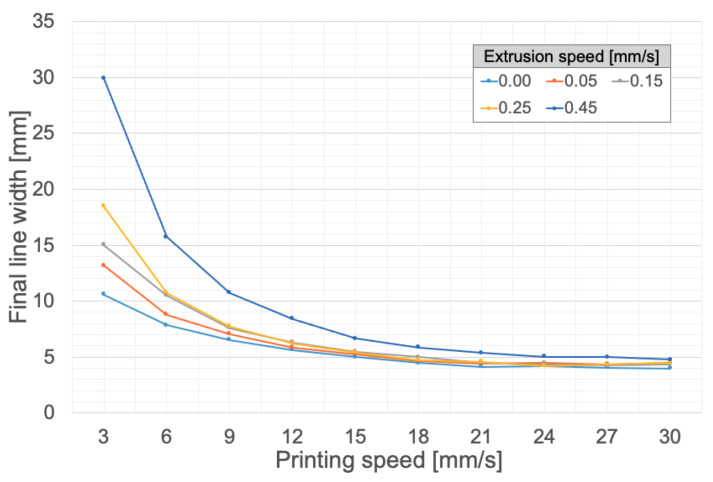
Influence of extrusion speed and printing speed on the final line width. Silicone: Ecoflex 00-20. Needle diameter: 1.6 mm. Bed temperature: 90 °C.

The same testing procedure was then used to determine the influence of the extrusion speed. In this case, instead of performing the test for four temperatures, it was performed for five extrusion speeds at a fixed temperature of 90 °C, since such a temperature (see Figure 3) yields smaller final widths and hence the finest details while printing. The obtained results are plotted in Figure 4.

Figure 4 demonstrates that a higher extrusion speed leads to a larger final line width. This result is expected, since a higher extrusion speed corresponds to a higher volumetric flow rate and thus to a greater amount of deposited material, which results in a larger final width. Here again, the gaps between different extrusion speeds are larger at lower printing speeds and, for a printing speed of 30 mm/s, a minimum line width is reached where all extrusion speeds yield line widths falling in a close range between 4 and 5 mm. Figure 4 also highlights the possibility of printing at an extrusion speed equal to zero, which corresponds to a spontaneous extrusion by gravity (as detailed in Section 2.3) and which actually leads to the smallest final widths of the printed lines.

#### 3.1.2. Influence of Silicone Viscosity and Needle Diameter on the Final Line Width

**Figure 5:**
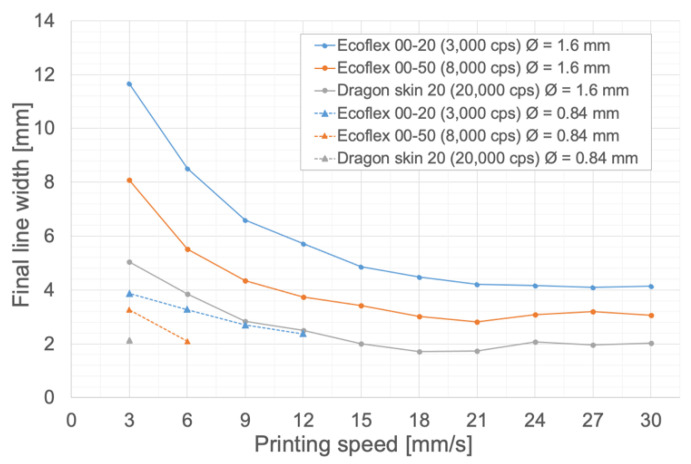
Influence of silicone viscosity and needle diameter on the final line width. Extrusion speed: 0 mm/s. Bed temperature: 90 °C.

To determine the influence of silicone viscosity on the final line width, the series of lines used in Section 3.1.1 was printed for the silicones Ecoflex 00-20, Ecoflex 00-50 and Dragon Skin 20 (listed in Table 1). Furthermore, to determine the influence of the needle diameter, the printings of the three types of silicone were performed for both 1.6 mm and 0.84 mm needle diameters while keeping the extrusion speed fixed at 0 mm/s and the bed temperature at 90 °C. The corresponding prints can be seen in Figure S3, and the final line widths, measured using the ImageJ software, are shown in Figure 5.

As observed from Figure 5, a higher silicone viscosity yields a smaller final line width, since a more viscous silicone will spread less on the printing bed before polymerization. Furthermore, the reduction of the needle diameter drastically reduces the final line width due to the reduction in material flow and, in the cases of the highest printing speeds, there is not even line formation because there is not enough flow for continuous material deposition (this can be observed in the three bottom images in Figure S3).

#### 3.1.3. Influence of Printing Height and Elapsed Time on the Final Line Width

For the sake of brevity, the corresponding results are reported in the Supplementary Information (see Sections S4 and S5 and Figures S4–S6). The printing height (i.e., the distance between the syringe tip and printing bed, here tested at 1 mm and 3 mm) does not affect the observed final line width. In contrast, it can be observed from the results that the elapsed printing time—from the moment Part A and Part B are mixed—has a mild impact on the final line width and that the installation of the syringe in the printer generates an increased flow rate at the beginning of the print before recovering the nominal value. Hence, if the line width needs to be precisely controlled, a waiting time of 45 s should be observed before launching the print so as to attain steady state flow. Additionally, for long prints, the increase in viscosity over time should be considered.

### 3.2. Printing of Silicone Geometric Shapes

In the previous sections, the final line width has been not only evaluated as a function of printing parameters (i.e., printing speed, extrusion speed and bed temperature), but also as a function of user-oriented parameters (i.e., silicone viscosity and syringe diameter). Keeping the silicone preparation time and syringe installation constant, the focus of the next sections is to determine the influence of the printing parameters involved when printing geometric shapes, i.e., the infill pattern and infill density. In the following, the tests were conducted with Ecoflex 00-20 and a 1.6 mm-diameter needle for a fixed printing speed of 30 mm/s, an extrusion speed of 0 mm/s, and a bed temperature of 90 °C. Note, however, that the proposed approach can be generalized to any printing parameters as long that they have been characterized. Here the focus was on squares and discs, since these shapes are involved in the targeted magnetoactive surface.

#### 3.2.1. Influence of Infill Density and Infill Pattern on the Printing of a Square

For a 1.6 mm-diameter needle, a printing speed of 30 mm/s, an extrusion speed of 0 mm/s and a bed temperature of 90 °C, the final width of Ecoflex 00-20 printed lines was ~4 mm. Since the silicone spreads 2 mm on either side of the printed line’s center, it is necessary—to avoid both over- or under-extrusion—that the infill lines have a separation between 2 mm and 4 mm, where at 2 mm there is complete overlap between two adjacent lines and where at 4 mm there is no overlap between two adjacent lines.

**Figure 6:**
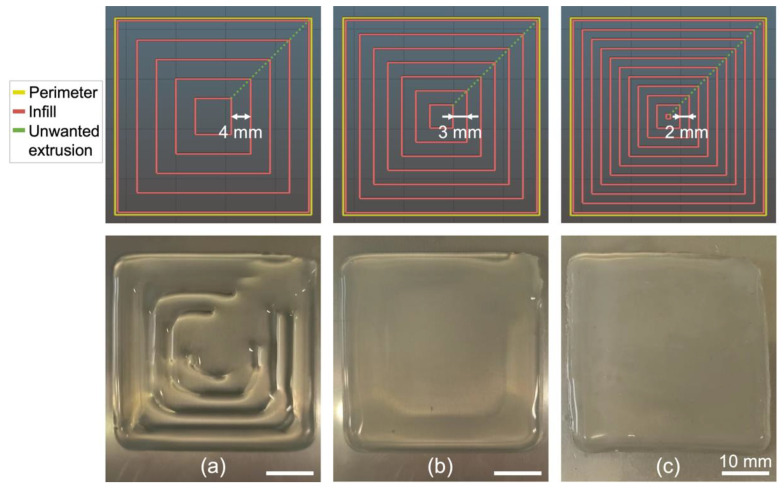
Tests to determine the optimal distance between infill lines in the case of a 40 mm × 40 mm square with a concentric infill pattern. (**Top**) Printing paths generated in Slic3r. (**Bottom**) Corresponding prints for (**a**) 13% infill density (4 mm line separation); (**b**) 18% infill density (3 mm line separation); and (**c**) 27% infill density (2 mm line separation). Silicone: Ecoflex 00-20. Needle diameter: 1.6 mm. Printing speed: 30 mm/s. Extrusion speed: 0 mm/s. Bed temperature: 90 °C.

The G-codes for the infill patterns (accounting for infill density) were obtained using the Slic3r software in the case of 40 mm × 40 mm squares. Since the magnetoactive surfaces are intended to be thin, the printed squares were designed to have only one printed layer. Three concentric infill patterns were first tested with infill densities of 13%, 18% and 27% corresponding to infill line separations of 4 mm, 3 mm and 2 mm, respectively. The infill patterns generated on Slic3r for each setting as well as the resulting printed squares are presented in Figure 6.

During the printing process, the perimeter (yellow line in Figure 6) is printed first. Then, the needle moves inwards to print the inner square and proceeds outwards to print the next square until it reaches the perimeter again. Unwanted extrusion (due to spontaneous extrusion by gravity, see Section 2.3) appears as a line connecting the top right corners of all squares and is highlighted in green in Figure 6. Note that it is only visible in the case of under-extrusion (13% infill).

A 4 mm line separation (13% infill) clearly leads to a square with under-extrusion, since the square is not completely filled (Figure 6a). With a 3 mm line separation (18% infill), no gaps between lines are observed but the outer region of the square appears thicker than the inner region (Figure 6b). In contrast, the 2 mm line separation (27% infill density) results in a complete square infill with neither over- nor under-extrusion (Figure 6c). For this last print, the overall size of the obtained square is ~42 mm × 42 mm, which is larger than the input geometry. This limited overall dimensional accuracy is expected due to the spread of the silicone during extrusion. However, the focus of this section is to obtain a good infill quality, and control of the overall size of the printed shape can be achieved by compensation, i.e., by imposing smaller dimensions to the designed input geometry.

To test whether other types of infill patterns may result in a better printed square, three other patterns (Archimedean chords, rectilinear and gyroid, see Figure S7) were tested while maintaining a distance of 2 mm between the infill lines. The other infill patterns led to undulations at the edges (or sometimes irregular thickness). Hence, the concentric pattern with an infill density of 27% yields the printing of a silicone square with good surface quality and dimensional accuracy, while the gyroid infill pattern with 50% infill density can be considered the second-best result obtained.

#### 3.2.2. Influence of Infill Pattern on the Printing of a Disc

**Figure 7:**
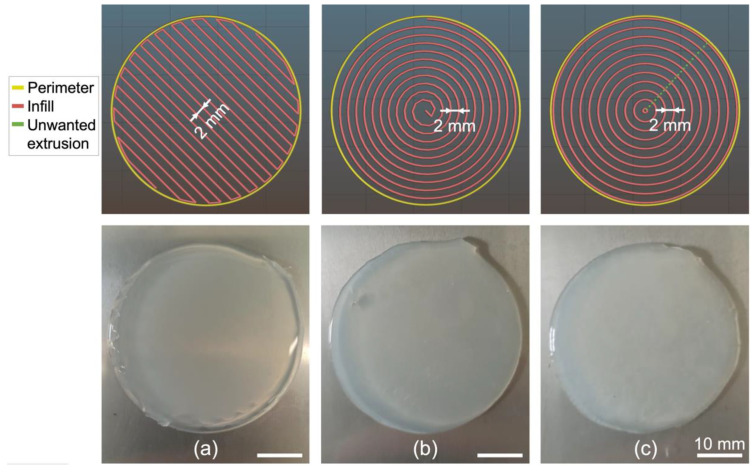
Tests to determine the optimal infill pattern in the case of a 40 mm diameter disc with an infill density adjusted to obtain a 2 mm line separation. (**Top**) Printing paths generated in Slic3r. (**Bottom**) Corresponding prints for (**a**) rectilinear with 27% infill density; (**b**) Archimedean chords with 30% infill density; and (**c**) concentric with 27% infill density. Silicone: Ecoflex 00-20. Needle diameter: 1.6 mm. Printing speed: 30 mm/s. Extrusion speed: 0 mm/s. Bed temperature: 90 °C.

Considering the results obtained regarding the infill density in the case of the square, only the effect of the type of pattern was tested in the case of the disc. Three settings were tested: rectilinear (with 27% infill density), Archimedean chords (with 30% infill density), and concentric (with 27% infill density), with all infill densities corresponding to a distance of 2 mm between infill lines. The infill patterns generated on Slic3r for each setting as well as the resulting printed discs are presented in Figure 7.

The best disc infill pattern among those tested was the concentric one with an infill density of 27% (Figure 7c), while the other infill patterns led again to undulations at the edges. Therefore, in both studied cases (square and disc), one can conclude that the concentric pattern produces a higher filling quality. This may indicate that this pattern has a good potential for printing closed shapes with more complex geometries using LAM, since the process relies on the natural spreading of the liquid on the bed surface to fill the printed body. Note that for the disc, similarly to the printed square, the printed shape shows a final dimension that is larger than the input geometry with an overall diameter of ~42 mm.

### 3.3. Printing of a Heterogenous Magnetoactive Surface

In this section, the printing and testing of a heterogeneous magnetoactive surface is discussed in more detail. In Section 3.2, the optimal parameters for printing a square and a disc separately have been determined. However, since the targeted magnetoactive surface is composed of a silicone square with four embedded magnetic discs, it is also necessary to verify if the conclusions drawn in the case of the printing of closed geometries still hold when printing a square with holes. The printing of the four discs is then addressed before determining the most appropriate approach for printing the heterogeneous magnetoactive layer.

#### 3.3.1. Influence of Infill Pattern on the Printing of a Square with Holes

The considered square was 40 mm × 40 mm. It bore four holes 17 mm in diameter and distributed in a square pattern so that they were 2 mm away from each other and from the borders of the square. Since the top magnetoactive layer had to be stiffer than the matrix, Ecoflex 00-50 silicone was used instead of Ecoflex 00-20 because it has greater stiffness than the latter, and because it is easier to prepare than Dragon Skin 20 due to its lower viscosity.

**Figure 8:**
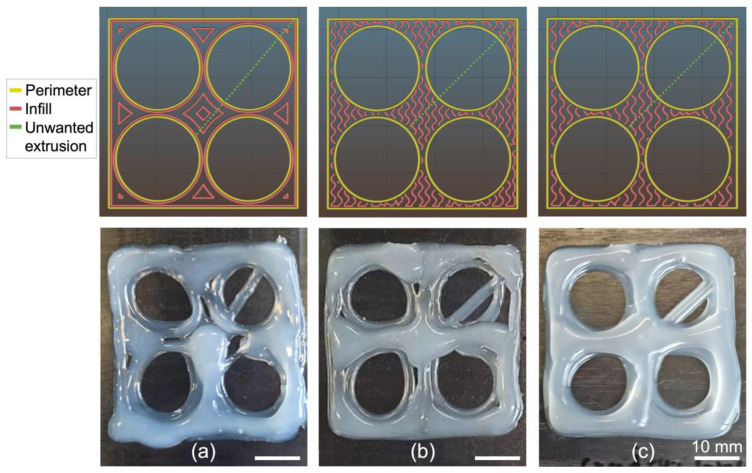
(**Top**) Printing paths generated in Slic3r. (**Bottom**) Corresponding prints of (**a**) a concentric pattern with 32% infill density and printing speed of 30 mm/s; (**b**) a gyroid pattern with 65% infill density and printing speed of 30 mm/s; and (**c**) a gyroid pattern with 56% infill density and printing speed of 10 mm/s. Silicone: Ecoflex 00-50. Needle diameter: 1.6 mm. Extrusion speed: 0 mm/s. Bed temperature: 90 °C.

Section 3.2.1 suggests that the best infill pattern for printing a square is the concentric one with an infill density corresponding to a spacing between the infill lines equal to half of the line width obtained with the selected printing parameters. Thus, the concentric pattern for the square with holes was first considered with an infill density of 32%. This corresponds to a line separation of 1.525 mm since the final line width is 3.05 mm for Ecoflex 00-50 silicone printed with: a 1.6 mm diameter needle, a printing speed of 30 mm/s, an extrusion speed of 0 mm/s and a bed temperature of 90 °C (see Figure 5). The printing path generated in Slic3r is presented in Figure 8a along with the corresponding print. It can be observed in Figure 8a that the concentric infill pattern is very different from those shown in Figure 6. It also leads to several displacements incompatible with continuous gravity extrusion: some regions of the square show excessive material deposition, especially at the bottom and center, while other regions have holes.

Seeking a more uniform material deposition, the gyroid infill pattern was tested since it presented the second-best result in terms of surface and dimensional quality and was associated with uniform displacements well-adapted to the LAM process (see Section S6 of the Supplementary Information). All the other printing parameters were maintained as above, except for the infill density that was set at 65% to enforce a line separation of 1.525 mm in the case of the gyroid pattern. The printing path generated in Slic3r, as well as the corresponding print, are presented in Figure 8b. For the square with holes, the print with the gyroid infill pattern gives somewhat better results than the concentric pattern, since it generates a less distorted shape with fewer defects. However, the print with the gyroid infill pattern still presents holes and non-uniform silicone depositions in some regions.

After further analysis, it was observed that, at high speeds, the silicone flow did not consistently follow the movement of the syringe when it changed direction frequently (this was not observed when printing straight lines). Therefore, it was necessary to reduce the printing speed and to adjust the spacing of the infill lines according to Figure 5, i.e., set an infill density of 56% for a line separation of 2.07 mm. The corresponding printing path generated in Slic3r is presented in Figure 8c along with the corresponding print. This time, there are no longer holes in the print and the silicone deposition is much more uniform throughout the printed shape, even though a step can be noticed between the perimeter lines and the rest of the infill. Printing without the perimeter was also tested (see Supplementary Materials Section S6). Figure S8 demonstrates that the step effect is no longer present (since there is no perimeter printed first) but the overall outlines of the shapes (i.e., the outer square and the inner circles) are strongly affected. Therefore, activating the perimeter in the slicing software ensures that the main outlines of the printed shapes are well-defined, especially in the case of non-closed shapes. The main drawback with activating the outline is the presence of unwanted extrusion when transitioning from the perimeter to the infill lines. Although this is not a major issue in the case of closed shapes (Figure 6 and Figure 7), this leads to an unwanted line of silicone going through a hole in the case of non-closed shapes (see Figure 8). However, considering the overall improvement achieved in print quality when activating the perimeter, this extra line is an acceptable compromise, since it can be easily removed using a scalpel. Finally, the overall size of the printed square is 42 mm × 42 mm and the average diameter of the holes is 12 mm.

#### 3.3.2. Printing of a Sequence of Four Discs

The discs to be printed were 17 mm in diameter and had a spatial arrangement identical to the holes distributed in the square discussed in Section 3.3.1.

**Figure 9:**
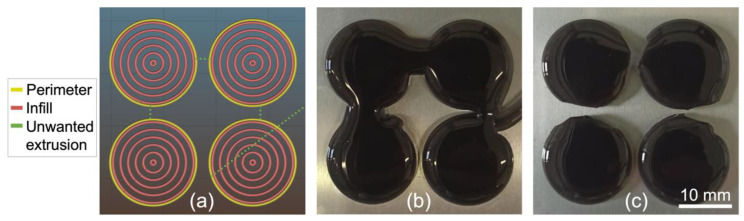
Printing of a sequence of four discs with a concentric infill pattern and 28% infill density. (**a**) Printing path generated in Slic3r; (**b**) corresponding print; and (**c**) print after removal of the unwanted extrusion. Silicone: Ecoflex 00-50 with black dye. Needle diameter: 1.6 mm. Printing speed: 10 mm/s. Extrusion speed: 0 mm/s. Bed temperature: 90 °C.

Section 3.2.2 shows that the best infill pattern for printing a disc is the concentric one, where the infill density still corresponds to a spacing between the infill lines equal to half of the line width obtained with the selected printing parameters. Considering these observations, the setting chosen for printing the series of discs was the concentric infill pattern with 28% infill density, corresponding to a separation of 2.07 mm between the infill lines for Ecoflex 00-50 printed with: a needle diameter of 1.6 mm, a printing speed of 10 mm/s, an extrusion speed of 0 mm/s and a bed temperature of 90 °C. The printing path generated in Slic3r, as well as the corresponding print, are presented in Figure 9. Note that in this test, a black dye was added to the silicone.

The selected settings guarantee a good dimensional and surface quality for the discs, but the main problem encountered is the occurrence of unwanted extrusion by gravity, which results in four extra lines of silicone on the printing surface. This gravity extrusion cannot be avoided when printing a sequence of shapes, even if they are closed. As discussed previously, these lines can be cut manually with a scalpel after printing, and even after manual cutting, the discs retain a good dimensional and surface quality (Figure 9c). In particular, the average diameter of the final discs is 17 mm. It is relatively easy here to cut the lines manually without great dimensional and shape loss because the diameter of the printed discs is large. However, this is not true for smaller discs (below 12 mm), for which manual cutting becomes much more detrimental and cumbersome, as discussed in the Supplementary Information (Figure S9).

#### 3.3.3. Printing of a Heterogeneous Magnetoactive Layer and Final Assembly

To print the heterogeneous magnetoactive layer, two approaches can be envisioned: (1) print the square with holes first and then print the discs within the holes, and (2) print the discs first and then the square with holes around the discs. In the first approach, the diagonal formed in the upper right circle by unwanted gravity deposition (Figure 8c) can be easily removed manually. However, the major disadvantage here is that the unwanted lines that arise during the printing of the discs (Figure 9b) are deposited on top of the square, resulting in a bonding between these lines that are composed of magnetic material and the square formed by pure silicone (see Figure S10). In contrast, the second approach has the advantage that the lines of unwanted deposition, formed during the printing of the discs, can be easily cut before carrying out the printing of the square. However, if no action is taken, unwanted deposition by gravity when printing the square with holes creates a silicone diagonal on top of the upper right magnetic disc. Although this is less of a problem because this will not affect the intended distribution of magnetic material in the layer, it can be easily avoided by placing a plastic cover on top of the upper right disc before printing the square with holes (see Figure S10).

**Figure 10:**
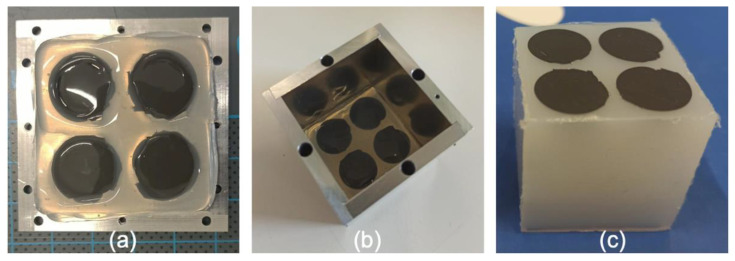
(**a**) 3D printed heterogeneous magnetic layer obtained with the printing parameters described in the previous sections and placed at the bottom of a mold; (**b**) mold after assembly of the walls; and (**c**) final 40 mm × 40 mm × 40 mm system composed of a heterogeneous magnetic layer resting on a soft non-magnetic substrate.

Hence, the second approach was retained for printing the heterogeneous magnetic layer. The discs were composed of a mixture of Ecoflex 00-50 silicone and iron particles (7.6 vol%) and the printing of the discs was carried out according to the parameters found in Section 3.3.2. Note that, in general, the addition of particles has an impact on the viscosity of the blend. However, the low particle content considered here was not observed to significantly affect the printing quality even though the parameters for pure silicone were employed. If higher particle contents were to be eventually considered, the printing parameter study conducted in Section 3.1 would have to be repeated for each high-concentration blend. The printing of the squares with holes was carried out with the printing parameters found in Section 3.3.1, and the resulting heterogeneous magnetoactive layer is presented in Figure 10a. After printing, the layer was placed at the bottom of a mold on which walls were subsequently installed. Once assembled (Figure 10b), the mold was filled with the low-stiffness Ecoflex 00-10 silicone and placed in an oven at 70 °C for 2 h to cure. Upon unmolding, the stiffer heterogeneous magnetoactive layer was fully bonded to a soft non-magnetic substrate (see [48] regarding the quality of the adhesion between layer and substrate) so that the system measured 40 mm × 40 mm × 40 mm overall, as shown in Figure 10c. The thickness of the layer was measured to be 2.2 mm.

### 3.4. Testing under Magnetic Field and Comparison to a 2D Numerical Simulation

**Figure 11:**
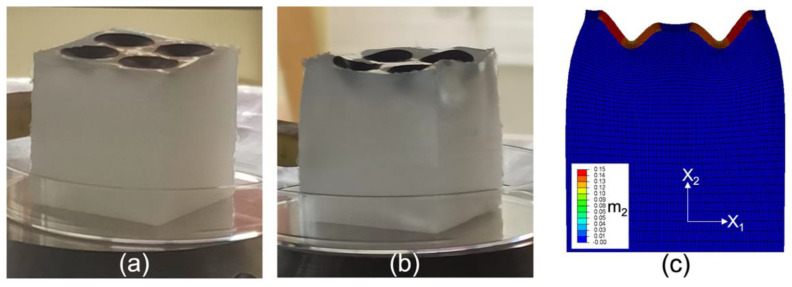
3D-printed stiff heterogeneous magnetic layer resting on a soft non-magnetic substrate submitted to a uniform magnetic field perpendicular to the layer (and pointing upwards), and showing 3D surface patterns under an applied field of (**a**) 0.4 T and (**b**) 0.8 T. (**c**) A 2D finite element simulation showing the inwards movement of the stiff magnetic discs into the soft non-magnetic substrate, where m_2_ (A/µm) is the magnetization in the X_2_-direction under an applied field of 0.4 T and is plotted on the deformed configuration.

The system obtained in Section 3.3.3 was then tested under a uniform magnetic field in an electromagnet described in [48], with the field direction perpendicular to the layer. Images of the system under a 0.4 T and a 0.8 T magnetic field pointing upwards are presented in Figure 11a,b, respectively. At 0.4 T, the magnetic discs exhibit an inwards movement into the soft non-magnetic substrate, thereby creating cavities on the surface of the cube. Upon increase of the magnetic field to 0.8 T, the magnetic discs dip further inwards into the substrate, forming even larger cavities that now yield a global undulation of the top surface of the cube. Hence, the proposed system yields truly 3D surface patterns upon application of a magnetic field. After reversing the polarity of the field, the deformation of the discs continues to form cavities dipping into the soft substrate. This is somewhat expected: MREs exhibit symmetric mechanical responses to magnetic fields with different polarities since they are not magnetized permanently.

A preliminary 2D finite element calculation was performed using Abaqus and a user-element subroutine presented in [47] and in [49]. The modeling used the constitutive laws and parameters discussed in [49]. More specifically, the mechanical behavior of the substrate was modeled as a standard neo-Hookean incompressible material with shear modulus μ = 3 kPa. The mechanical behavior of the top layer was also modeled by a neo-Hookean incompressible law with shear modulus μ = 10 kPa, and the magnetic susceptibility of the discs was set to χ = 2/3. As explained in [49], the air around the system was also meshed. The motion of the air was constrained to follow the deformation of the boundary of the deforming solid. This constitutes the most efficient modeling of the air, allowing the system to reach large strains without convergence issues and without any effect of the air upon the mechanical stiffness of the system. The magnetization (along the field direction) under a uniformly applied magnetic field of 0.4 T is presented in Figure 11c. It is plotted on the deformed contours of the system (consisting of the substrate, the top non-magnetic film, and the magnetic discs), and these contours capture qualitatively well the behavior observed experimentally. Although this is only a preliminary two-dimensional simulation, it still reveals the deformation modes observed in the experiments. A full three-dimensional study is a subject for future work.

It appears that the behavior of the magnetic discs is driven not only by boundary conditions but also by the instability-type response already discussed in the context of Figure 1b and in the work of Psarra et al. [49]. In this regard, the observed behavior can be reasonably explained in 2D using Figure 11c by considering the disc as a plane-strain (i.e., large out-of-plane dimension) layer. As the field is applied, the magnetic layer tries to rotate in order to align with the applied field (which is perpendicular to the layer). This rotation is simply due to the compass effect, but it is restrained by the substrate. The presence of corners (this is the case in both two and three dimensions) at the edges of the layer creates a strong concentration of the magnetic field. Thus, the corners first start moving slightly upwards. In turn, this creates a non-uniform magnetic distribution which pushes the central part of the disc downwards in an effort to rotate the entire layer. However, the presence of the substrate penalizes this motion in such a way that a finite wavelength dip is created. Moreover, due to the freestanding lateral boundaries of the specimen, the resistance is lower near the edges of the substrate than it is at the center of the substrate, explaining why the outer edge of the magnetic layer rises more than the edge located at the center of the substrate. Finally, the fact that the discs move inwards independently of the field direction is directly related to the symmetric mechanical response of MREs under magnetic fields of opposite polarity. More simply, the MREs under study do not retain a permanent magnetization upon removal of the magnetic field, and thus the response is independent of the magnetic direction [50].

## 4. Conclusions

In this article, a general method for printing pure silicone via LAM is proposed, starting with the evaluation of the width of printed lines as a function of the following parameters: printing speed, extrusion speed, bed temperature, syringe needle diameter, print height, elapsed printing time and silicone viscosity. This work demonstrates that, once such behavior of the silicone has been understood, from the silicone line width for a given set of parameters, it is possible to determine what infill patterns and infill densities should be selected in order to print shapes with good surface and dimensional qualities. Although the infill parameters are straightforward and transposable for closed shapes (concentric pattern with a density that is directly related to the line width), it is shown that the printing strategy must be carefully studied in the case of open shapes and adapted to the continuous deposition that is specific to LAM. Finally, it is demonstrated that the printing of a heterogeneous layer, composed of four magnetoactive discs embedded in a pure silicone square, is possible via LAM.

LAM certainly has drawbacks including the need for removal of unwanted lines due to continuous deposition, limited complexity of printable shapes, limited dimensional accuracy, low in-plane resolution (4 mm minimal line size compared to ~100 µm for FFF and DIW and ~20 µm for SLA and DLP) as well as high layer thickness (~2 mm compared to ~20 µm for FFF and DIW and <10 µm for SLA and DLP). However, it is the only technique that allows the printing of magneto-active blends that remain very soft since the only fillers added to the silicone—already very soft—are the fillers directly related to the sought functional properties. DIW could provide a better-controlled extrusion, but it requires additional fillers (leading to increased hardness of the resulting composite) to impart the ink with shear-thinning properties. In contrast, FFF can only print hard to semi-hard polymers and SLA and DLP semi-hard to soft polymers. Offering the possibility to print very soft functional silicones is especially important for the rapid manufacturing of morphing layers in which the pattern drawn with the functional blend is directly linked to the surface profile obtained under actuation. A standard technique such as molding, as it requires manufacturing a mold for each pattern, would render the exploration of a large pattern space very burdensome.

Finally, the layer proposed in this work, when affixed to a softer silicone substrate, yields a system that produces truly three-dimensional surface patterns upon application of a magnetic field. Hence, this study proves that LAM is a suitable avenue to pursue for printing surfaces that exhibit spatially heterogeneous magnetic particle content, and thereby produce surfaces with different magnetic patterns and shapes. In turn, this opens the door to the generation of on-demand, reversible 3D surface patterns for a wide range of applications ranging from haptics to camouflage to differential cell growth, as recently demonstrated by Moreno-Mateos [51]. Further work is under way to experimentally measure the obtained 3D surface profiles in order to compare them with 3D numerical predictions for magneto-active discs containing different particle contents. The development of a 3D model precisely capturing the magneto-mechanical couplings is indeed of great interest for the design of more complex on-demand magneto-activated patterns.

## Figures and Tables

**Table 1:** Properties of the silicones used in this study.

Silicone	Working Time (min)	Mixing Ratio	Mixed Viscosity (cps)	Shore Hardness (ASTM D-2240)
Ecoflex 00-20	30	1A:1B	3000	00-20
Ecoflex 00-50	18	1A:1B	8000	00-50
Ecoflex 00-10	30	1A:1B	14,000	00-10
Dragon Skin 20	25	1A:1B	20,000	20A

## Data Availability

The raw/processed data required to reproduce these findings cannot be shared at this time as the data also form part of an ongoing study.

## Supplementary Materials

The following are available online at www.mdpi.com/xxx/s1.pdf. Figure S1: Resulting printed body when feeding silicone into the syringe by suction. Figure S2: Test conducted to determine the influence of printing speed, extrusion speed and bed temperature on the final line width. Silicone: Ecoflex 00-20. Needle diameter: 1.6 mm. Extrusion speed: 0.05 mm/s. Bed temperature: 70 °C. Figure S3: Tests to determine the influence of silicone viscosity and needle diameter on the final line width.Silicone: Ecoflex 00-20. Extrusion speed: 0 mm/s. Bed temperature: 90 °C. Figure S4: Influence of printing height on the final line width. Silicone: Ecoflex 00-20. Needle diameter: 1.6 mm. Bed temperature: 90 °C. Extrusion speed: 0 mm/s. Figure S5: Test for determining the influence of the elapsed time on the final line width. Silicone: Ecoflex 00-20. Needle diameter: 1.6 mm. Bed temperature: 90 °C. Printing speed: 30 mm/s. Extrusion speed: 0.05 mm/s. Figure S6: Influence of the elapsed printing time on the final line width. Silicone: Ecoflex 00-20. Needle diameter: 1.6 mm. Printing speed: 30 mm/s. Extrusion speed: 0.05 mm/s. Bed temperature: 90 °C. The black dotted line corresponds to the linear fit running through the last four points of each preparation time. Figure S7: Tests to determine the optimal infill pattern in the case of a 40 mm × 40 mm square with an infill density adjusted to obtain a 2 mm line separation. (Top) Printing paths generated in Slic3r. (Bottom) Corresponding prints for (a) Archimedean chords with 30% infill density, (b) rectilinear with 27% infill density, (c) gyroid with 50% infill density, and (d) concentric with 27% infill density. Silicone: Ecoflex 00-20. Needle diameter: 1.6 mm. Printing speed: 30 mm/s. Extrusion speed: 0 mm/s. Bed temperature: 90 °C. Figure S8: Tests to determine the influence of the perimeter on the printing quality of a 40 mm × 40 mm square with 17 mm diameter holes. (Top) Printing paths generated in Slic3r. (Bottom) Corresponding prints for (a) a concentric pattern with 40% infill density, (b) a rectilinear pattern with 35% infill density, and (c) a gyroid pattern with 56% infill density. Silicone: Ecoflex 00-50. Needle diameter: 1.6 mm. Printing speed: 10 mm/s. Extrusion speed: 0 mm/s. Bed temperature: 90 °C. Figure S9: Test to determine the minimum diameter achievable for the printed discs. Sequence of six discs printed from left to right with a diameter of 18 mm, 15 mm, 12 mm, 9 mm, 6 mm and 4 mm (a) as printed, and (b) after manual cutting with a scalpel. Silicone: Ecoflex 00-50 with black dye. Needle diameter: 1.6 mm. Printing speed: 10 mm/s. Extrusion speed: 0 mm/s. Bed temperature: 90 °C. Concentric infill pattern with 28% infill density. Figure S10: Tests to determine the best printing order in the case of a 40 mm × 40 mm silicone square with four embedded 17 mm diameter discs from (a) printing of the square with holes followed by the printing of the discs within the holes, (b) printing of the discs, removal of the unwanted lines, and placement of a cover before further printing, and (c) printing of the square with holes around the discs. Silicone: Ecoflex 00-50 (with black dye for the discs). Needle diameter: 1.6 mm. Printing speed: 10 mm/s. Extrusion speed: 0 mm/s. Bed temperature: 90 °C. Concentric infill pattern with 28% infill density for the discs. Gyroid infill pattern with 56% infill density for the square with holes.

## References

[1] Liu T., Xu Y. (2019). Magnetorheological Elastomers: Materials and Applications. Smart and Functional Soft Materials.

[2] Wu S., Hu W., Ze Q., Sitti M., Zhao R. (2020). Multifunctional Magnetic Soft Composites: A Review. Multifunct. Mater..

[3] Lucarini S., Hossain M., Garcia-Gonzalez D. (2022). Recent Advances in Hard-Magnetic Soft Composites: Synthesis, Characterisation, Computational Modelling, and Applications. Compos. Struct..

[4] Ginder J.M., Clark S.M., Schlotter W.F., Nichols M.E. (2002). Magnetostrictive Phenomena in Magnetorheological Elastomers. Int. J. Mod. Phys. B.

[5] Martin J.E., Anderson R.A., Read D., Gulley G. (2006). Magnetostriction of Field-Structured Magnetoelastomers. Phys. Rev. E.

[6] Bodelot L., Voropaieff J.-P., Pössinger T. (2018). Experimental Investigation of the Coupled Magneto-Mechanical Response in Magnetorheological Elastomers. Exp. Mech..

[7] Guan X., Dong X., Ou J. (2008). Magnetostrictive Effect of Magnetorheological Elastomer. J. Magn. Magn. Mater..

[8] Stepanov G.V., Kramarenko E.Y., Semerenko D.A. (2013). Magnetodeformational Effect of the Magnetoactive Elastomer and Its Possible Applications. J. Phys. Conf. Ser..

[9] Ginder J.M., Nichols M.E., Elie L.D., Clark S.M., Wereley N.M. (2000). Controllable-Stiffness Components Based on Magnetorheological Elastomers. Smart Structures and Materials 2000: Smart Structures and Integrated Systems.

[10] Farshad M., le Roux M. (2004). A New Active Noise Abatement Barrier System. Polym. Test..

[11] Moon F.C., Pao Y.-H. (1968). Magnetoelastic Buckling of a Thin Plate. J. Appl. Mech..

[12] Gerbal F., Wang Y., Lyonnet F., Bacri J.-C., Hocquet T., Devaud M. (2015). A Refined Theory of Magnetoelastic Buckling Matches Experiments with Ferromagnetic and Superparamagnetic Rods. Proc. Natl. Acad. Sci. USA.

[13] Lum G.Z., Ye Z., Dong X., Marvi H., Erin O., Hu W., Sitti M. (2016). Shape-Programmable Magnetic Soft Matter. Proc. Natl. Acad. Sci. USA.

[14] Kim J., Chung S.E., Choi S.-E., Lee H., Kim J., Kwon S. (2011). Programming Magnetic Anisotropy in Polymeric Microactuators. Nat. Mat..

[15] Hu W., Lum G.Z., Mastrangeli M., Sitti M. (2018). Small-Scale Soft-Bodied Robot with Multimodal Locomotion. Nature.

[16] Xu T., Zhang J., Salehizadeh M., Onaizah O., Diller E. (2019). Millimeter-Scale Flexible Robots with Programmable Three-Dimensional Magnetization and Motions. Sci. Robot..

[17] Zhao R., Kim Y., Chester S.A., Sharma P., Zhao X. (2019). Mechanics of Hard-Magnetic Soft Materials. J. Mech. Phys. Solids.

[18] Li Y., Li J., Li W., Du H. (2014). A State-of-the-Art Review on Magnetorheological Elastomer Devices. Smart Mater. Struct..

[19] Ahamed R., Choi S.-B., Ferdaus M.M. (2018). A State of Art on Magneto-Rheological Materials and Their Potential Applications. J. Intel. Mater. Syst. Struct..

[20] Bira N., Dhagat P., Davidson J.R. (2020). A Review of Magnetic Elastomers and Their Role in Soft Robotics. Front. Robot. AI.

[21] Farahani R.D., Dubé M., Therriault D. (2016). Three-Dimensional Printing of Multifunctional Nanocomposites: Manufacturing Techniques and Applications. Adv. Mater..

[22] Truby R.L., Lewis J.A. (2016). Printing Soft Matter in Three Dimensions. Nature.

[23] Falahati M., Ahmadvand P., Safaee S., Chang Y.-C., Lyu Z., Chen R., Li L., Lin Y. (2020). Smart Polymers and Nanocomposites for 3D and 4D Printing. Mater. Today.

[24] Palmero E.M., Rial J., de Vicente J., Camarero J., Skårman B., Vidarsson H., Larsson P.-O., Bollero A. (2018). Development of Permanent Magnet MnAlC/Polymer Composites and Flexible Filament for Bonding and 3D-Printing Technologies. Sci. Technol. Adv. Mater..

[25] Lin C., Lv J., Li Y., Zhang F., Li J., Liu Y., Liu L., Leng J. (2019). 4D-Printed Biodegradable and Remotely Controllable Shape Memory Occlusion Devices. Adv. Funct. Mater..

[26] Qi S., Fu J., Xie Y., Li Y., Gan R., Yu M. (2019). Versatile Magnetorheological Plastomer with 3D Printability, Switchable Mechanics, Shape Memory, and Self-Healing Capacity. Compos. Sci. Technol..

[27] Cao X., Xuan S., Sun S., Xu Z., Li J., Gong X. (2021). 3D Printing Magnetic Actuators for Biomimetic Applications. ACS Appl. Mater. Interfaces.

[28] Ji Z., Yan C., Yu B., Wang X., Zhou F. (2017). Multimaterials 3D Printing for Free Assembly Manufacturing of Magnetic Driving Soft Actuator. Adv. Mater. Interfaces.

[29] Lu L., Baynojir Joyee E., Pan Y. (2017). Correlation Between Microscale Magnetic Particle Distribution and Magnetic-Field-Responsive Performance of Three-Dimensional Printed Composites. J. Micro Nano Manuf..

[30] Lantean S., Barrera G., Pirri C.F., Tiberto P., Sangermano M., Roppolo I., Rizza G. (2019). 3D Printing of Magnetoresponsive Polymeric Materials with Tunable Mechanical and Magnetic Properties by Digital Light Processing. Adv. Mater. Technol..

[31] Gratson G.M., Xu M., Lewis J.A. (2004). Direct Writing of Three-Dimensional Webs. Nature.

[32] Lewis J.A. (2006). Direct Ink Writing of 3D Functional Materials. Adv. Funct. Mater..

[33] Kim Y., Yuk H., Zhao R., Chester S.A., Zhao X. (2018). Printing Ferromagnetic Domains for Untethered Fast-Transforming Soft Materials. Nature.

[34] Zhu P., Yang W., Wang R., Gao S., Li B., Li Q. (2018). 4D Printing of Complex Structures with a Fast Response Time to Magnetic Stimulus. ACS Appl. Mater. Interfaces.

[35] Kim Y., Parada G.A., Liu S., Zhao X. (2019). Ferromagnetic Soft Continuum Robots. Sci. Robot..

[36] Roh S., Okello L.B., Golbasi N., Hankwitz J.P., Liu J.A.-C., Tracy J.B., Velev O.D. (2019). 3D-Printed Silicone Soft Architectures with Programmed Magneto-Capillary Reconfiguration. Adv. Mater. Technol..

[37] Zhang Y., Wang Q., Yi S., Lin Z., Wang C., Chen Z., Jiang L. (2021). 4D Printing of Magnetoactive Soft Materials for On-Demand Magnetic Actuation Transformation. ACS Appl. Mater. Interfaces.

[38] Bastola A.K., Paudel M., Li L. (2018). Development of Hybrid Magnetorheological Elastomers by 3D Printing. Polymer.

[39] Bastola A.K., Paudel M., Li L. (2019). Line-Patterned Hybrid Magnetorheological Elastomer Developed by 3D Printing. J. Intell. Mater. Syst. Struct..

[40] Hinton T.J., Hudson A., Pusch K., Lee A., Feinberg A.W. (2016). 3D Printing PDMS Elastomer in a Hydrophilic Support Bath via Freeform Reversible Embedding. ACS Biomater. Sci. Eng..

[41] Plott J., Shih A. (2017). The Extrusion-Based Additive Manufacturing of Moisture-Cured Silicone Elastomer with Minimal Void for Pneumatic Actuators. Addit. Manuf..

[42] Liravi F., Toyserkani E. (2018). A Hybrid Additive Manufacturing Method for the Fabrication of Silicone Bio-Structures: 3D Printing Optimization and Surface Characterization. Mater. Des..

[43] Luis E., Pan H.M., Sing S.L., Bajpai R., Song J., Yeong W.Y. (2020). 3D Direct Printing of Silicone Meniscus Implant Using a Novel Heat-Cured Extrusion-Based Printer. Polymers.

[44] LiQ 320–3D Printing from Liquid Silicone. https://www.innovatiq.com/en/products/3d-printers/liq-320/.

[45] Feng J., Xuan S., Lv Z., Pei L., Zhang Q., Gong X. (2018). Magnetic-Field-Induced Deformation Analysis of Magnetoactive Elastomer Film by Means of DIC, LDV, and FEM. Ind. Eng. Chem. Res..

[46] Raikher Y.L., Stolbov O.V., Stepanov G.V. (2008). Deformation of a Circular Ferroelastic Membrane in a Uniform Magnetic Field. Tech. Phys..

[47] Dorn C., Bodelot L., Danas K. (2021). Experiments and Numerical Implementation of a Boundary Value Problem Involving a Magnetorheological Elastomer Layer Subjected to a Nonuniform Magnetic Field. J. Appl. Mech..

[48] Psarra E., Bodelot L., Danas K. (2017). Two-Field Surface Pattern Control via Marginally Stable Magnetorheological Elastomers. Soft Matter..

[49] Psarra E., Bodelot L., Danas K. (2019). Wrinkling to Crinkling Transitions and Curvature Localization in a Magnetoelastic Film Bonded to a Non-Magnetic Substrate. J. Mech. Phys. Solids.

[50] Danas K., Kankanala S.V., Triantafyllidis N. (2012). Experiments and Modeling of Iron-Particle-Filled Magnetorheological Elastomers. J. Mech. Phys. Solids.

[51] Moreno-Mateos M.A., Gonzalez-Rico J., Nunez-Sardinha E., Gomez-Cruz C., Lopez-Donaire M.L., Lucarini S., Arias A., Muñoz-Barrutia A., Velasco D., Garcia-Gonzalez D. (2022). Magneto-Mechanical System to Reproduce and Quantify Complex Strain Patterns in Biological Materials. Appl. Mat. Today.

