# Supplementary material for: Towards 4D Printing of Very Soft Heterogeneous Magnetoactive Layers for Morphing Surface Applications via Liquid Additive Manufacturing
Source: Polymers (Basel). 2022 Apr 21;14(9):1684. doi: 10.3390/polym14091684 (PMC9105100; doi:10.3390/polym14091684)
Supplement: Supplementary file 1 [file polymers-14-01684-s001.zip › polymers-1664709-supplementary.pdf]

**SUPPLEMENTARY INFORMATION for**

# Towards 4D Printing of Very Soft Heterogeneous Magnetoactive Layers for Morphing Surface Applications via Liquid Additive Manufacturing

Lucas Brusa da Costa Linn[1], Kostas Danas[1], Laurence Bodelot[1]

[1] Solid Mechanics Laboratory (LMS), CNRS, École Polytechnique, Institut Polytechnique de Paris, 91128 Palaiseau, France

## 1 Syringe feeding

During the sample preparation process, feeding the syringe with the silicone blend can be carried out in two ways: by pouring or by suction. As shown in **Figure S1**, feeding the syringe by suction led to a large number of bubbles in the printed body as air was mixed again with the degassed silicone during the suction.

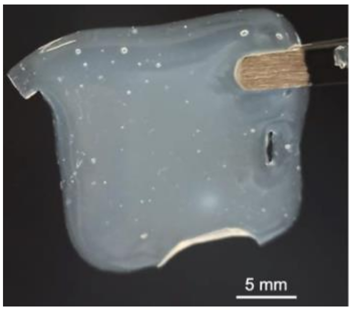


**Figure S1.** Resulting printed body when feeding silicone into the syringe by suction.

## 2 Influence of bed temperature, printing speed, and extrusion speed on the final line width

To determine the influence of bed temperature and printing speed on the final width of the lines, a series of lines was printed at a fixed temperature, where the printing speed was reduced at each line as illustrated in **Figure S2**. Note that in these tests, the tool path was programmed manually, i.e., without the aid of the slicer, and all samples were prepared following the steps presented in Section 2.3 of the main article.

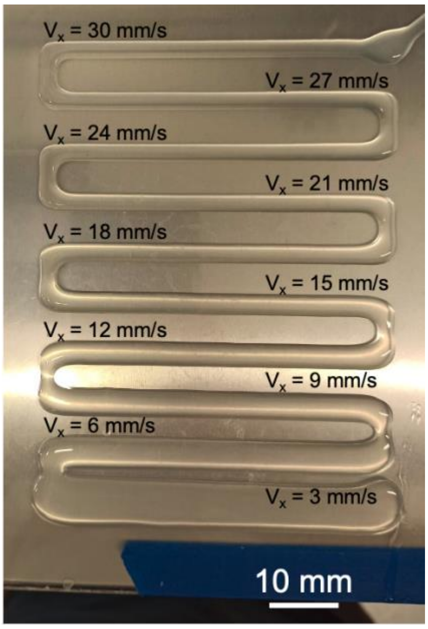


**Figure S2**. Test conducted to determine the influence of printing speed, extrusion speed and bed temperature on the final line width. Silicone: Ecoflex 00-20. Needle diameter: 1.6 mm. Extrusion speed: 0.05 mm/s. Bed temperature: 70 °C.

## 3 Influence of silicone viscosity and needle diameter on the final line width

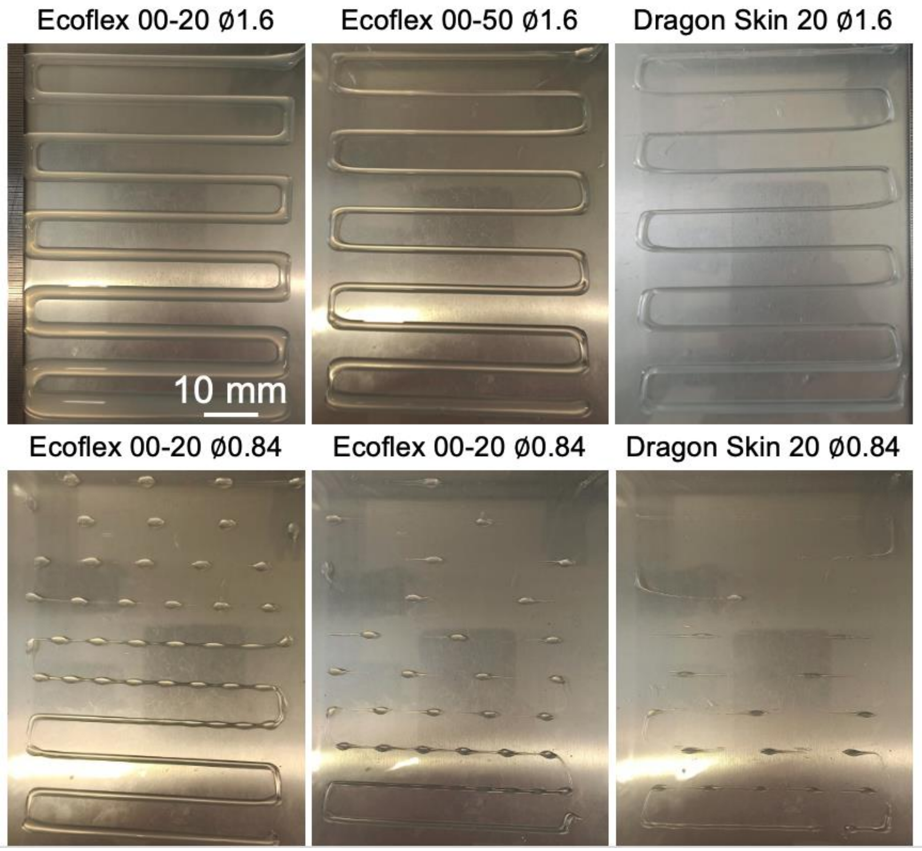


**Figure S3.** Tests to determine the influence of silicone viscosity and needle diameter on the final line width. Silicone: Ecoflex 00-20. Extrusion speed: 0 mm/s. Bed temperature: 90 °C.

## 4 Influence of printing height on the width of printed Ecoflex 00-20 silicone lines

To determine the influence of the printing height (i.e., the distance between the syringe tip and the printing bed) on the final width of the lines, a series of lines was printed for two heights: 1 mm and 3 mm, with the extrusion speed set at 0 mm/s (gravity extrusion) and the bed temperature set at 90 °C (**Figure S4**).

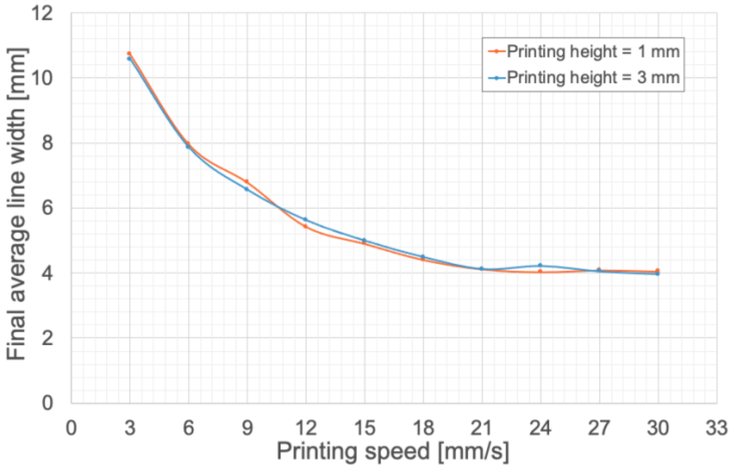


**Figure S4.** Influence of printing height on the final line width. Silicone: Ecoflex 00-20. Needle diameter: 1.6 mm. Bed temperature: 90 °C. Extrusion speed: 0 mm/s.

Even though it can be assumed that a greater printing height leads to a greater spread of the silicone on the printing bed, **Figure S4** shows that the final widths of printed lines are virtually unchanged by a change in the printing height.

## 5 Influence of the elapsed time on the width of printed Ecoflex 00-20 silicone lines

Since the silicone that is fed into the syringe has already been mixed, the curing process starts occurring during the printing, though at a very slow rate since it requires 24 h to obtain full curing at room temperature. Nevertheless, in order to assess the effect of the elapsed time—from the moment Part A and Part B are mixed—on the width of the printed lines, a series of successive lines was printed, where the only variable was the time between each line. Hence in this case, the printing speed, the extrusion speed and the bed temperature were fixed at 30 mm/s, 0.05 mm/s and 90 °C, respectively. At each line, a command was written for the machine to wait for 20 s before starting the next line. Since the length of each line was 100 mm, the time to print each line was approximately 3 s. Adding 1 s to perform a 30 mm shift on the Y-axis for positioning of the next line led to a total of 24 s between each line. Deposition by gravity during the displacement on the Y-axis and the 20 s wait generated a continuous deposition of material in the form of large circles on the printing surface, as shown in **Figure S5**.

The aforementioned test was repeated for three different preparation times: (1) 20 s of mixing and 1.5 min of degassing (total of 110 s), (2) 25 s of mixing and 2.75 min of degassing (total of 190 s), and (3) 30 s of mixing and 4 min of degassing (total of 270 s). Finally, 30 s was added to these preparation times to account for pouring the silicone in the syringe and for installing the syringe in the printer before launching the print.

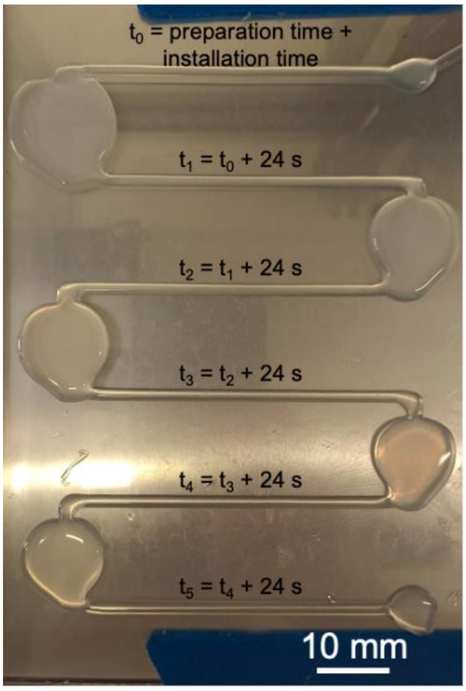


**Figure S5.** Test for determining the influence of the elapsed time on the final line width. Silicone: Ecoflex 00-20. Needle diameter: 1.6 mm. Bed temperature: 90 °C. Printing speed: 30 mm/s. Extrusion speed: 0.05 mm/s.

The results obtained after measuring the width of the lines using the ImageJ software are reported in **Figure S6** to demonstrate the effect of the elapsed time on the printing process. Since the silicone viscosity increases over time, the final line width decreases when the time elapsed before printing increases. The behavior is almost linear if the first two printed lines for each preparation time are disregarded. Indeed, when the plunger is sunk into the syringe for installation in the printer, air is pushed down, which creates extra pressure and an increased flow rate at the beginning of the print before recovering the nominal value. A linear fit run through the last four points of each preparation time (dotted line in **Figure S6**) leads to a slope of 0.004 (with $R^2 = 0.97$), which means a 4 µm decrease in printed line width can be expected per elapsed second. For an initial line width of 4.6 mm, this would amount to a 10.4% decrease in width after 2 min of printing.

Hence, if the line width needs to be precisely controlled, a waiting time of 45 s should be observed before launching the print so as to attain a steady state flow. Additionally, for long prints, the increase of viscosity over time should be considered.

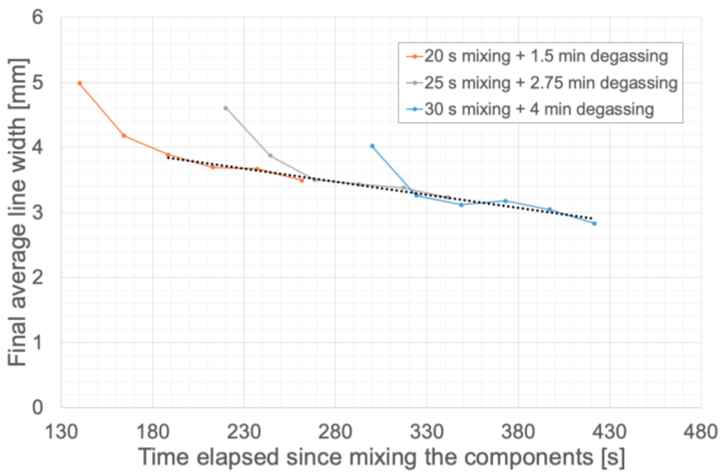


**Figure S6.** Influence of the elapsed printing time on the final line width. Silicone: Ecoflex 00-20. Needle diameter: 1.6 mm. Printing speed: 30 mm/s. Extrusion speed: 0.05 mm/s. Bed temperature: 90 °C. The black dotted line corresponds to the linear fit running through the last four points of each preparation time.

# 6 Influence of different types of infill patterns on the printing of a square

For all the tested patterns, the infill density was adjusted so that a distance of 2 mm was maintained between the infill lines.

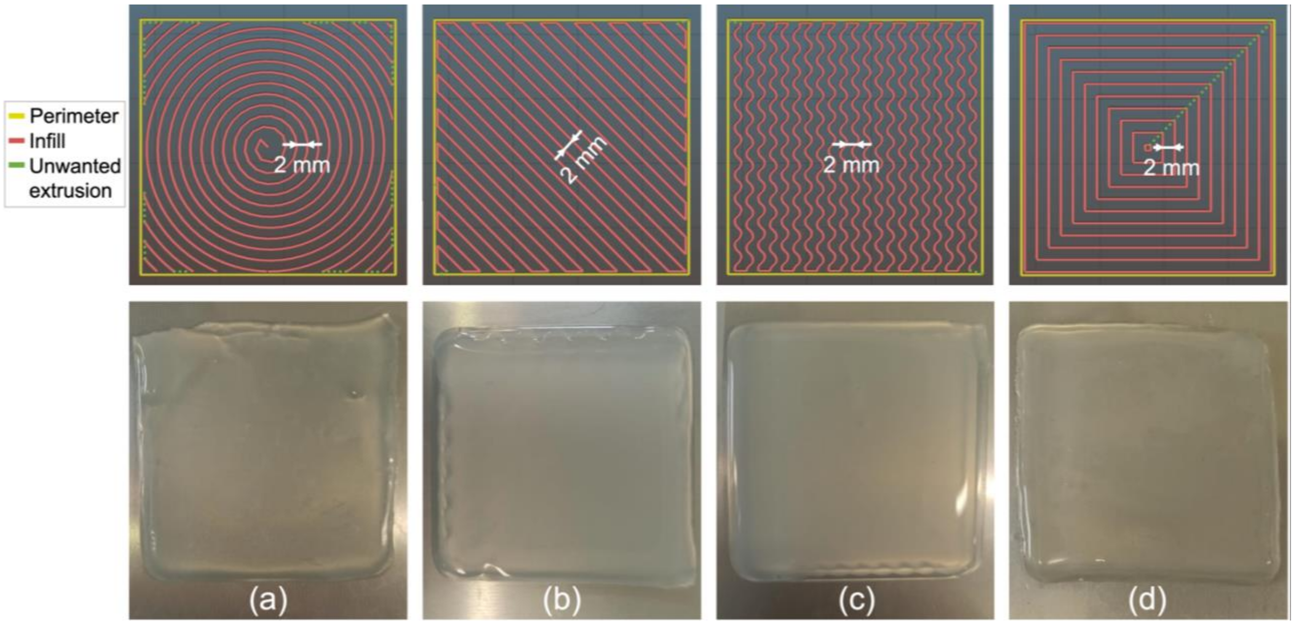


**Figure S7.** Tests to determine the optimal infill pattern in the case of a 40 mm x 40 mm square with an infill density adjusted to obtain a 2 mm line separation. (**Top**) Printing paths generated in Slic3r. (**Bottom**) Corresponding prints for (**a**) Archimedean chords with 30% infill density, (**b**) rectilinear with 27% infill density, (**c**) gyroid with 50% infill density, and (**d**) concentric with 27% infill density. Silicone: Ecoflex 00-20. Needle diameter: 1.6 mm. Printing speed: 30 mm/s. Extrusion speed: 0 mm/s. Bed temperature: 90 °C.

Except for the concentric infill pattern (**Figure S7**d), all the other printing patterns tested (**Figure S7**a-c) lead to prints that exhibit undulations at the edges or holes, although it can be observed that the gyroid infill pattern with 50% infill density also presents a rather good surface and dimensional quality. It thus can be considered the second-best result obtained after the concentric pattern.

## 7 Influence of the perimeter line on the printing of a square with holes

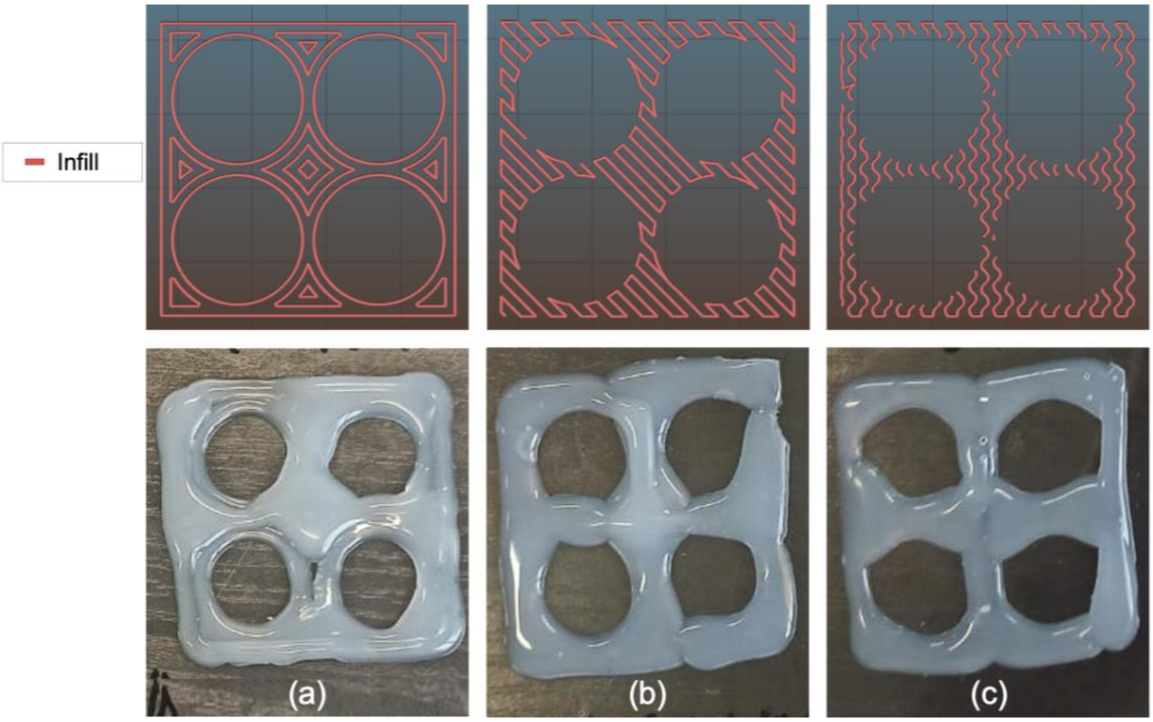


**Figure S8.** Tests to determine the influence of the perimeter on the printing quality of a 40 mm x 40 mm square with 17 mm diameter holes. (**Top**) Printing paths generated in Slic3r. (**Bottom**) Corresponding prints for (**a**) a concentric pattern with 40% infill density, (**b**) a rectilinear pattern with 35% infill density, and (**c**) a gyroid pattern with 56% infill density. Silicone: Ecoflex 00-50. Needle diameter: 1.6 mm. Printing speed: 10 mm/s. Extrusion speed: 0 mm/s. Bed temperature: 90 °C.

Although there is no unwanted extrusion by gravity when printing without a perimeter, the outlines of the corresponding circles are not well defined, presenting variable diameters and shapes that later impair the printing of the discs. Therefore, printing the square with holes—or non-closed shapes in general—without a perimeter is not satisfactory.

## 8 Determination of the minimum diameter achievable for the printed discs

The minimum diameter of the discs that can be printed is limited by two factors: the spread of silicone on the printing surface and the subsequent need for manual cutting of unwanted lines formed by gravity deposition. Hence, the diameter must be large enough to allow a precise manual cutting that does not affect the size and shape of the discs. To determine the minimum printable diameter, discs with six different diameters (18 mm, 15 mm, 12 mm, 9 mm, 6 mm and 4 mm) were printed in a sequence. Like the test for the sequence of four discs, Ecoflex 00-50 silicone with black dye was used with a needle diameter of 1.6 mm, a printing speed of 10 mm/s, an extrusion speed of 0 mm/s, a bed temperature of 90 °C and a concentric infill pattern with 28% infill density. The series of discs before and after cutting the unwanted lines formed by gravity extrusion are shown in **Figure S9**.

In **Figure S9**, one can observe that diameters smaller than 12 mm begin to lose their circular shape, acquiring a more elliptical shape. As the diameter of the disc becomes smaller, it gets closer to the width of the line, and the distinction between the circular shape and the line becomes difficult. In addition, for diameters smaller than 12 mm, performing a manual cutting to obtain discs with the desired shapes and dimensions is impractical since manual cutting with a scalpel is not precise enough to transform the elliptical shape into a circular one. Thus, with the chosen printing parameters, the smallest possible diameter that offers a relatively good dimensional quality is approximately 12 mm.

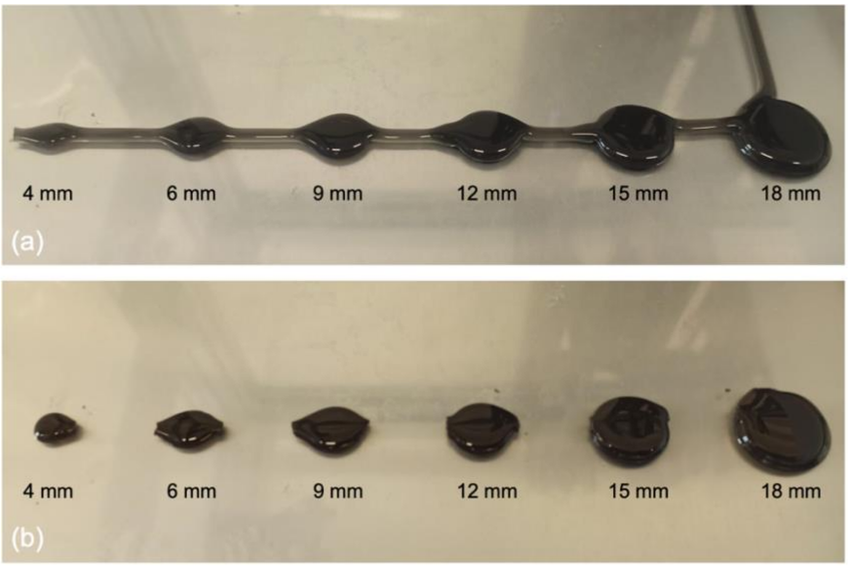


**Figure S9.** Test to determine the minimum diameter achievable for the printed discs. Sequence of six discs printed from left to right with a diameter of 18 mm, 15 mm, 12 mm, 9 mm, 6 mm and 4 mm (**a**) as printed, and (**b**) after manual cutting with a scalpel. Silicone: Ecoflex 00-50 with black dye. Needle diameter: 1.6 mm. Printing speed: 10 mm/s. Extrusion speed: 0 mm/s. Bed temperature: 90 °C. Concentric infill pattern with 28% infill density.

## 9 Influence of printing order in the case of a silicone square with four embedded discs

To print the heterogeneous magnetoactive layer, two approaches can be considered: (1) print first the square with holes and then print the discs within the holes, and (2) print first the discs and then the square with holes around the discs. Both these options were tested with Ecoflex 00-50 (with black dye for the discs), a needle diameter of 1.6 mm, a printing speed of 10 mm/s, an extrusion speed of 0 mm/s and a bed temperature of 90 °C. The concentric infill pattern with an infill density of 28% was used for the sequence of four discs, and the gyroid infill pattern with an infill density of 56% was used for the square with holes. In (1), the diagonal formed in the upper right circle by unwanted gravity deposition (see Figure 8 in the main article) was removed manually before printing the discs; however, the unwanted lines that arise during the printing of the discs (see Figure 9b in the main article) bonded with the square formed by pure silicone (**Figure S10**a). In (2), the lines of unwanted deposition formed during the printing of the discs were cut before carrying out the printing of the square, and a plastic cover was placed on top of the right disc before printing the square with holes (**Figure S10**b). As a result, a heterogeneous layer, composed of two different materials respecting their intended shapes, was obtained (**Figure S10**c).

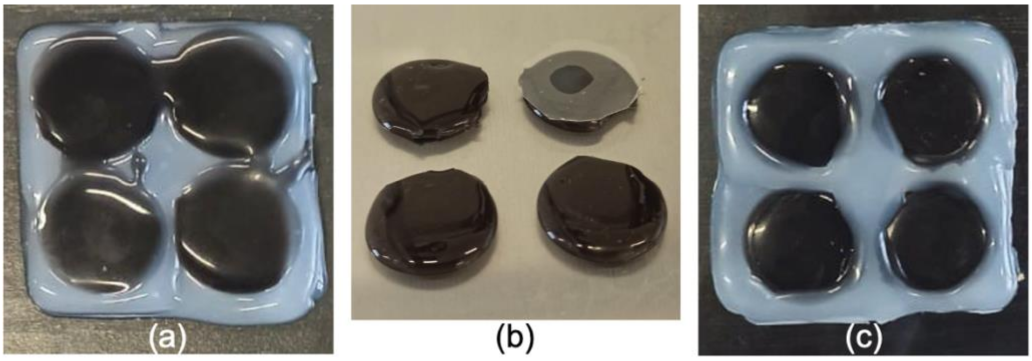


**Figure S10.** Tests to determine the best printing order in the case of a 40 mm x 40 mm silicone square with four embedded 17 mm diameter discs from (**a**) printing of the square with holes followed by the printing of the discs within the holes, (**b**) printing of the discs, removal of the unwanted lines, and placement of a cover before further printing, and (**c**) printing of the square with holes around the discs. Silicone: Ecoflex 00-50 (with black dye for the discs). Needle diameter: 1.6 mm. Printing speed: 10 mm/s. Extrusion speed: 0 mm/s. Bed temperature: 90 °C. Concentric infill pattern with 28% infill density for the discs. Gyroid infill pattern with 56% infill density for the square with holes.
